# Pandemic preparedness of dentists against coronavirus disease: A Saudi Arabian experience

**DOI:** 10.1371/journal.pone.0237630

**Published:** 2020-08-19

**Authors:** Khalifa S. Al-Khalifa, Rasha AlSheikh, Abdullah S. Al-Swuailem, Muneera S. Alkhalifa, Mahmoud H. Al-Johani, Saud A. Al-Moumen, Zainab I. Almomen

**Affiliations:** 1 Preventive Dental Sciences Department, College of Dentistry, Imam Abdulrahman Bin Faisal University, Dammam, Saudi Arabia; 2 Restorative Dental Sciences Department, College of Dentistry, Imam Abdulrahman Bin Faisal University, Dammam, Saudi Arabia; 3 Department of Periodontics and Community Dentistry, College of Dentistry, King Saud University, Riyadh, Saudi Arabia; 4 Restorative Dental Department, Prince Sultan Military Medical City, Riyadh, Saudi Arabia; 5 Restorative Dental Department, East Jeddah Hospital, Ministry of Health, Jeddah, Saudi Arabia; 6 Dental Department, Ministry of Health, Dammam, Saudi Arabia; 7 Royal College of Surgeons, Dublin, Ireland; Charles Sturt University, AUSTRALIA

## Abstract

**Background:**

Dental offices are among the highest risk for transmission of the COVID-19, having the potential to transmit the virus via routine dental procedures. This cross-sectional study assessed the preparedness and perception of infection control measures against the COVID-19 pandemic by dentists in Saudi Arabia.

**Materials and methods:**

This online survey addressed the impact and perception of the COVID-19 pandemic on dental practice in Saudi Arabia. The questionnaire comprised 26 closed-ended questions. Descriptive statistics included frequency distributions with percentages. In addition, the significance between the different demographic variables and questions about dentists’ perception of the COVID-19 pandemic was tested using the Chi-square test.

**Results:**

COVID-19 management in dental clinics varied in terms of adherence to the Ministry of Health (MOH) guidelines. Dental clinics’ screening questionnaire for patients showed good adherence (67%), while the lowest agreement was detected with the question on the existence of an airborne infection in the isolation room (15%). Almost two-thirds of the respondents agreed that the dental reception area adopted the proper COVID-19 preventive measures. Greatest accord was observed in their answers on questions about dentists’ perception of the COVID-19 pandemic, ranging from 64%–89%. In addition, there were statistically significant differences in questions about the perception of dentists towards the COVID-19 pandemic by different demographic variables such as age and years of work experience (p < 0.05).

**Conclusion:**

The response of most dentists regarding the preparedness and perception of infection control measures against the COVID-19 pandemic was positive. Dental clinics need to adhere more to the MOH recommendations in preparedness of their facilities or by educating their dentists and staff.

## Introduction

The concept of "Infection control" has received considerable attention from dental professionals and organizations. With the emergence of new infectious diseases or sources of infection, preventive measures have escalated to higher levels according to the latest recommendations by the Centers for Disease Control and Prevention (CDC) and other global protection agencies [1–3]. Dental professionals have always been taught on protecting themselves and their patients from potential pathogens. However, the severe acute respiratory syndrome coronavirus 2 (SARS-CoV-2) infection, better known as coronavirus disease (COVID-19), has brought a new, unanticipated challenge to dental professionals [4]. The truth of the matter is that standard personal protective equipment (PPE) is not enough in cases of airborne infections such as COVID-19. The next level of infection control "transmitted–base precaution" should be taken into action by upgrading the PPE with materials such as unique masks (e.g., N-95), face protection or shield, gown, or coverall, head cover, and rubber boots [5–7].

Several scenarios of COVID-19 transmission have been explained, all agreed on droplet transmission, with an estimated spread substantially higher than that for seasonal influenza [8, 9]. Due to the nature of dental practice procedures which can generate a cloud of aerosol render dental offices to be among the highest risk categories for transmission of the COVID-19 [10–12]. For this reason, routine dental care has been suspended during the COVID-19 pandemic to aid in reducing the transmission of the infection [13–17]. Nevertheless, dentists are ethically obligated to provide emergency dental services to patients who need urgent care regardless of their health status [15–17]. It is the dental practitioner's moral and ethical responsibility to balance patients' needs and public health concerns.

The growing fear of COVID-19 infection transmission has mandated the development of new guidelines and recommendations by health authorities [5, 18]. The Ministry of Health (MOH) in Saudi Arabia has responded promptly to this outbreak by adopting the guidelines and recommendations of the World Health Organization (WHO) and CDC available to all dental practitioners on the MOH’s website [19, 20].

Despite the positive attitude of dental professionals in Saudi Arabia and proper practice of droplet and airborne infections in similar outbreaks [21], there seems to be a lack of knowledge and attitude towards "transmission–based precautions" in general by dental professionals, which calls for proper training [22]. None of the previous studies have examined the preparedness and perception towards combating the COVID-19 pandemic among dentists in Saudi Arabia. Therefore, this cross-sectional study aimed to assess dentists' preparedness and perception of infection control measures against the COVID-19 pandemic in Saudi Arabia.

## Materials and methods

### Survey instrument

An electronic survey of dental practitioners in Saudi Arabia was carried out from the May 23 to 31, 2020. The survey was adapted from several pre-validated surveys addressing the impact, perception and attitudes of dentist during the COVID-19 pandemic where the intra-class correlation ranged between 0.74 to 0.80 [23–25]. The questionnaire comprised a total of 26 closed-ended questions, and was designed using the SoGoSurveys^®^ software, which has been provided as a webpage link [26]. The questionnaire was divided into four sections: the first section of the survey solicited demographic and professional background information of the respondents. The second section of the survey covered questions about COVID-19 management in dental clinics. The third section of the survey covered questions about the COVID-19 preventive measures in the dental reception area. The fourth section of the survey covered questions about knowledge, practice, and attitudes of dentists towards the COVID 19 pandemic. Two of the authors (KK and AS) who are experts on the research subject reviewed the questionnaire and had a consensus on the validity of the questionnaire. The survey was then pretested on a group of 20 general dentists to gain feedback, and overall acceptability of the questionnaire was gotten along with minimal corrections based on their responses. In addition, the questionnaire was validated through intra-class correlation with a strong relation of 0.70. Ethical approval was granted since this was a survey-based study. The need for a consent by the participants was waived by the Ethical Committee of the College of Dentistry, Imam Abdulrahman Bin Faisal University as returned surveys meant that participants agreed to participate in the survey.

### Survey distribution

The sample size was calculated considering a 90% confidence level, 5% margin of error, expected dentist population of 10000 dentists according to latest statistics from the Saudi Commission for Health Specialties and a 50% response distribution, the minimum required sample size was calculated (http://www.raosoft.com/samplesize.html) to be 264.

An e-mail list of 1000 randomly chosen dental practitioners was obtained from the Saudi Commission for Health Specialties database. The random sample shared common demographic and professional characteristics with the dental workforce in Saudi Arabia. The survey was then distributed by e-mail, and a reminder was sent to all practitioners who did not respond to the first e-mail after 2–3 days from the initial e-mail sent.

### Data analysis

The data were entered in Microsoft Excel (2010) and transferred to IBM SPSS Statistics for Windows, version 22 (IBM Corp., Armonk, NY, USA) for statistical analysis. Descriptive statistics included frequency distributions with percentages. In addition, the significance between the different demographic variables such as age, gender, qualification, work experience in years, work setting of the main job and working hours per week, and questions about knowledge, practice, and attitude of dentists towards the COVID-19 pandemic was tested using the Chi-square test. Variables with more than two levels, such as age, work experience in years, and working hours per week, were dichotomized into two groups for ease of statistical interpretation. P-values < 0.05 were considered statistically significant.

## Results

Between May 23 and 31, 2020, 1 000 surveys were e-mailed to dental practitioners, 287 responses were returned, indicating a response rate of 28.7%.

Demographic data revealed that about 40% of the study population was aged 20–34 years. Over half of the respondents (55.7%) were males, over 60% were general dental practitioners, and one-third had less than five years of working experience. Practitioners from the Central region of Saudi Arabia constituted 40.4% of the respondents, followed by the Western region (27.2%) and the Eastern region (15.3%). The majority of respondents were working in public practice (60%), followed by private practice (27.2%). A large proportion of respondents (61%) worked 35–49 hours per week, followed by 20.6% of the respondents who reported working 20–34 hours per week (Table 1).

**Table 1:** Description of the demographic and professional characteristics of participants.

Characteristics	N (%)
**Age (in years)**	
20–34 yr	109 (38.0)
35–44 yr	96 (33.4)
45–54 yr	66 (23.0)
55–64 yr	16 (5.6)
**Gender**	
Male	160 (55.7)
Female	127 (44.3)
**Qualification**	
Consultant/Specialist	110 (38.3)
General dental practitioner	177 (61.7)
**Working experience (in years)**	
0–5 yr	87 (30.3)
6–10 yr	62 (21.6)
11–15 yr	38 (13.2)
> 16 yr	100 (34.8)
**Location of the main job**	
Central	116 (40.4)
Western	78 (27.2)
Eastern	44 (15.3)
Southern	39 (13.6)
Northern	10 (3.5)
**Work setting of the main job**	
Private	78 (27.2)
Public	172 (59.9)
Both (private & public)	18 (6.3)
Academic	19 (6.6)
**Working hours per week**	
1–19 hr	33 (11.5)
20–34 hr	59 (20.6)
35–49 hr	175 (61.0)
50 + hr	20 (7.0)
**Total**	**287**

Regarding questions about COVID-19 management in dental clinics, most respondents (65%) agreed that their dental clinic had a work plan (workflow) for COVID-19 patient screening and dental management. A similar percentage (67%) was observed in the COVID-19 screening questionnaire for patients. Regarding the isolation room for suspected COVID-19 patients, 46% of the respondents mentioned that it did not exist, and only 38% mentioned that it existed. The respondents were split in half (43% vs. 43%) about whether tele-screening was offered for patients before their dental visit. The next two questions were regarding COVID-19 preventive measures in the dental clinic, namely the existence of an airborne infection isolation room (AIIR) and an extra-oral suction (vacuum) system in the dental clinic. Most respondents said they both did not exist in the dental clinic they worked at (72% and 59%, respectively). About half of the respondents mentioned that their dental clinic did not offer proper COVID-19 management training sessions for their staff.

For the questions about the dental reception’s preventive measures against COVID-19, a vast majority of the respondents (92%) acknowledged that patients were required to take their body temperature before any dental procedure. About half of the respondents (47%) agreed that patients need to use an antiseptic mouth rinse before the dental procedure. The requirement of wearing a face mask in the waiting area was observed by 68% of the respondents. A similar percentage was noted by the respondents for patients' hand washing/sanitizing before going to the waiting area. Social distancing was practiced in the waiting area, as observed by 77% of the respondents.

**Fig 1:**
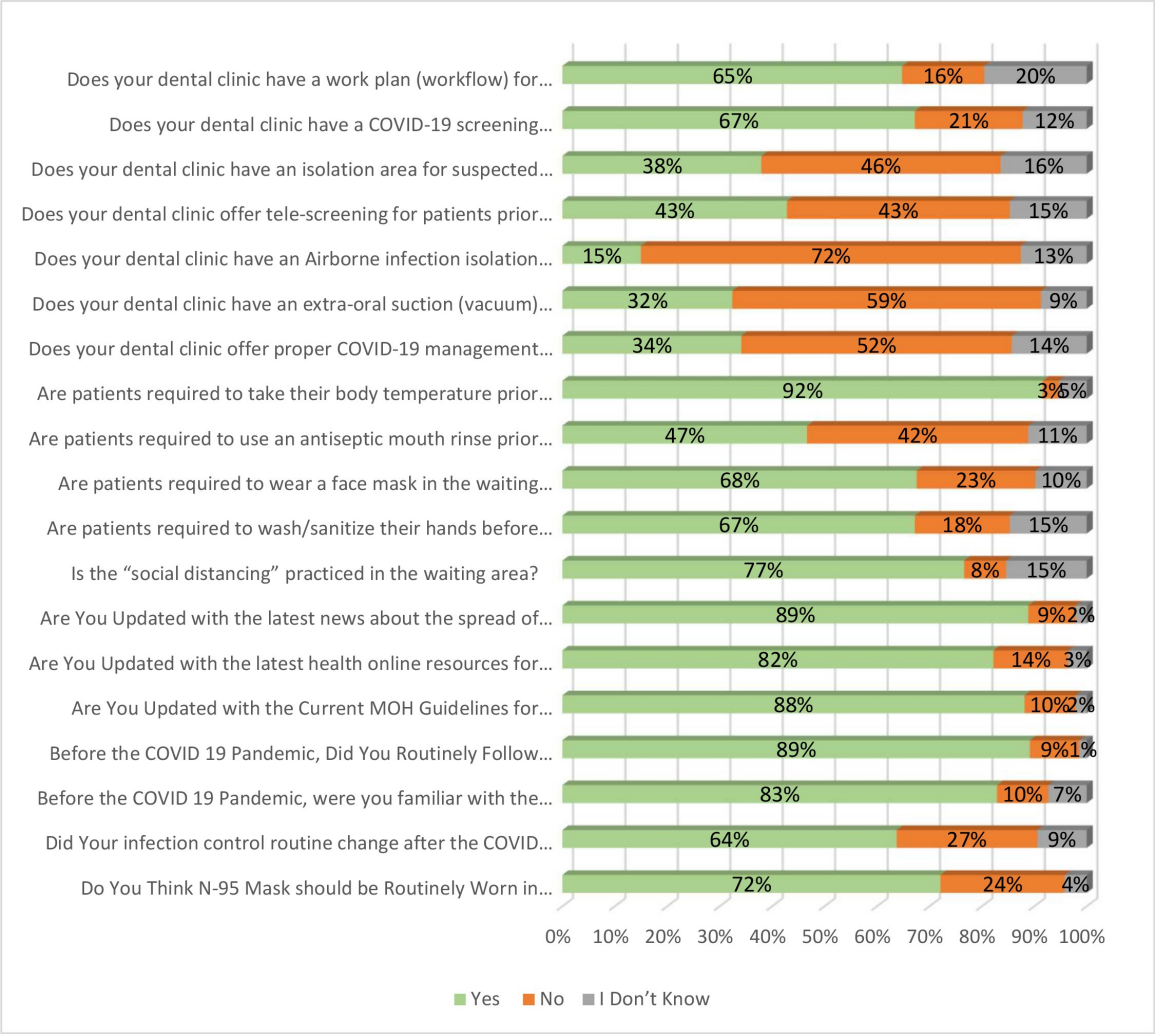
Responses of dentists’ preparedness and perception of infection control measures against the COVID-19 pandemic.

The last part of the questionnaire focused on the awareness and attitude of dentists towards the COVID-19 pandemic. Most of the respondents agreed on questions about the knowledge, practice, and attitude of dentists towards the COVID-19 pandemic. Of these, 89% were updated with the latest news about the spread of COVID-19. Of the respondents, 82% were updated on the latest health online resources for COVID-19; 88% of the respondents reported following the MOH guidelines for infection control regarding COVID-19. Before the COVID 19 Pandemic, 89% of the respondents routinely followed universal precautions of infection control for every patient. In addition, 83% of the respondents were familiar with the "transmission-based Precautions" for dental procedures. When asked whether their infection control routine changed after the COVID-19 pandemic, 64% concurred with that. The last question asked whether N-95 Mask should be routinely worn in dental practice as a new precaution, and 72% of the respondents agreed to that (Fig 1).

The next level involved bivariate analysis for questions about knowledge, practice, and attitude of dentists towards the COVID-19 pandemic by different demographic variables such as age, gender, qualification, working experience in years, work setting of the main job, and working hours per week. The age of participants had an effect on the knowledge about COVID-19, with older participants were more aware of the latest health online resources for COVID-19 (p = 0.043). Before the COVID 19 pandemic, older respondents reported that they routinely followed universal precautions of infection control for every patient (p = 0.015). On the other hand, younger respondents thought that the N-95 mask should be routinely worn in dental practice as a new precaution as compared to older respondents (p = 0.005).

As for the role of qualification in the knowledge, practice, and attitude of dentists towards the COVID-19 pandemic, two questions showed statistically significant differences between consultant/specialists and general dental practitioners, namely the latest news about the spread of the COVID-19 pandemic (90% vs. 88.1%, p = 0.021) and whether the infection control routine changed after the COVID-19 pandemic (73.6% vs. 57.6%, p = 0.023).

Only three questions about knowledge, practice, and attitude of dentists towards the COVID-19 pandemic showed statistically significant differences with work experience in years. Those who worked more than 11 years were more interested in being updated with the latest news about the spread of the COVID-19 pandemic (p = 0.008). Before the COVID 19 pandemic, those who worked for more than 11 years were more familiar with the "transmission-based Precautions" for dental procedures (p = 0.026). In addition, they admitted that their infection control routine changed after the COVID-19 pandemic (p = 0.006). Questions about knowledge, practice, and attitude of dentists towards the COVID-19 pandemic by working hours per week showed only two statistically significant differences. Respondents who worked over 35 hours per week reported being more updated with the latest health online resources for COVID-19 (p = 0.004). The term "Transmission-Based Precautions" was more familiar for those who worked over 35 hours per week (p = 0.031).

Gender showed no statistically significant differences in the questions about knowledge, practice, and attitude of dentists towards the COVID-19 pandemic, except for the question about whether the N-95 mask should be routinely worn in dental practice as a new precaution (p = 0.045). The same applies to work settings where only one question was statistically significant as more private practitioners reported they were aware of the term "Transmission-Based Precautions" (p = 0.049). (Table 2)

**Table 2:** Significant associations for questions about knowledge, practice, and attitude of dentists towards the COVID-19 pandemic by different demographic variables.

	Variable	Yes (%)	p-value
Are You Updated with the latest news about the spread of the COVID-19 Pandemic?	*Qualification*		
	Consultant/Specialist	99 (90.0)	0.021
	General dental practitioner	156 (88.1)	
Are You Updated with the latest health online resources for COVID-19?	*Age*		
	≤ 44 yr	162 (79.9)	0.043
	≥ 45 yr	74 (90.2)	
	*Years of experience*		
	≤ 10 yr	125 (83.9)	0.008
	≥ 11 yr	130 (94.2)	
	*Working hours per week*		
	≤ 34 hr	66 (71.7)	0.004
	≥ 35 hr	170 (87.2)	
Before the COVID 19 Pandemic, Did You Routinely Follow Universal Precautions of Infection Control for Every Patient?	*Age*		
	≤ 44 yr	176 (85.9)	0.015
	≥ 45 yr	80 (97.6)	
Before the COVID 19 Pandemic, were you familiar with the “Transmission-Based Precautions” for dental procedures?	*Years of experience*		
	≤ 10 yr	115 (77.2)	0.026
	≥ 11 yr	123 (89.1)	
	*Work setting of the main job*		
	Private	70 (89.7)	0.049
	Public	138 (80.2)	
	*Working hours per week*		
	≤ 34 hr	72 (78.3)	0.031
	≥ 35 hr	166 (85.1)	
Did your infection control routine change after the COVID 19 Pandemic?	*Qualification*		
	Consultant/Specialist	81 (73.6)	0.023
	General dental practitioner	102 (57.6)	
	*Years of experience*		
	≤ 10 yr	83 (55.7)	0.006
	≥ 11 yr	100 (63.8)	
Do you think N-95 mask should be routinely worn in dental practice as a new precaution?	*Age*		
	≤ 44 yr	158 (77.1)	0.005
	≥ 45 yr	49 (59.8)	
	*Gender*		
	Male	114 (71.2)	0.045
	Female	93 (73.2)	

## Discussion

Available evidence suggests that the complete control of COVID-19 infection is not possible in the foreseeable future until a vaccine is developed [27, 28]. This fact implies that managing this public health problem relies primarily on developing guidelines to prevent the transmission of COVID-19 infection. Thus, the success in containing this infection will mainly depend on the development and adherence to the set of guidelines and protocols proposed by national and international health organizations [13]. The findings of this study are alarming, as about one-third of sampled dentists reported that there was no work plan, or they were unaware that there was a work plan for COVID-19 patient screening and dental management in their practices. Proper channeling of patients using a clear work plan for COVID-19 was an essential element in guidelines proposed by some international health organizations [29, 30].

It has been recommended to use tele-screening for patients to avoid contact with patients who could be a source of infection to the dental office staff and other patients [18, 29, 31]. It is not surprising that more than half of the participating dentists in this study responded that their practices did not employ tele-screening for patients before their dental visits, as it requires extra-staff with special training to conduct such tasks. Giudice et al. reported that the use of tele-dentistry would allow patients to screen and monitor cases while limiting human contact, thus decreasing the risk for COVID-19 transmission [32]. Alternatively, dental practices may need to consider using questionnaire-based screening for patients before they are admitted to the dental office. In this study, about two-thirds of participating dentists indicated that their practice was using questionnaire-based screening for patients to identify high-risk patients.

The majority of participating dentists reported that their practices did not have an isolation area for suspected COVID-19 patients or airborne infection isolation rooms. This is in accordance with the general recommendations that limit contact with patients who may have COVID-19 infection [18]. Nonetheless, dental practices need special arrangements to manage urgent cases that mandate immediate intervention. This intervention, when needed, should be performed in a setting that prevents or minimizes transmission of COVID-19 infection to dental staff and other patients [33, 34]. One such procedure is using extra-suction beyond regular saliva suction. This device helps to reduce bacterial load and hazardous bio-aerosols during routine dental treatment [35, 36]. In our study, only one-third of the participating dentists indicated that extra-oral suction was used in their practice.

Over time, it is expected that dentists all over the world will adjust to the new reality that COVID-19 infection has changed the norms in dental practice and meet the new challenges of this severe pandemic. Part of this adjustment is that dentists need to be updated with new regulations and guidelines as the management of COVID-19 infection changes rapidly. In this study, only one-third of participating dentists reported that their practices offered COVID-19 management training sessions. This low percentage suggests that practicing dentists should take a leading role in educating themselves on policies that protect them, their staff, and patients from COVID-19 infection.

The vast majority of respondents (92%) indicated that patients must have their body temperatures measured before any dental procedure is performed. This measure of infection control has been reported in other studies. For example, Consolo et al. reported that practicing dentists in Italy will consider measuring the patient's temperature to minimize the likelihood of transmitting COVID-19 to dental staff and/or other patients [37]. In another study that surveyed 650 dentists in 30 countries, 81% of participating dentists indicated that measuring a patient's body temperature is mandatory before undertaking any dental procedure [23]. Furthermore, social distancing has been advocated as an essential measure to prevent the transmission of COVID-19 infection [33, 38]. In this survey, the results suggest that about three-quarters of participating dentists have indicated that reducing the number of patients in waiting areas, "so-called social distancing," is employed in their practices. Dentists have considered this method to be the most critical method to prevent the transmission of COVID-19 infection [37]. The survey findings suggest that other methods for preventing the transmission of COVID-19 infection, such as patients’ wearing a face mask in the waiting area and hand washing/sanitizing before going to the waiting area were less popular. The use of these two methods has been reported in other studies with comparable figures [25, 37]. On the other hand, almost half of the dentists (47%) preferred patients using antiseptic mouth rinse before dental treatment. Ahmad et al. (74%) and Canetti et al. (75%) reported that most (74%) dentists indicated that they did not practice the use of antiseptic mouth rinse before dental treatment during this pandemic [23, 25].

Dental consultants and specialists showed significantly higher awareness response (90%) as well as dentists with experience of eleven years or more (94.2%) regarding the latest news of the COVID-19 pandemic. Many studies have reported similar results, where higher awareness was observed among professionals with higher education and experience [39–42]. Dentists aged 45 years and older and those with longer working hours (≥ 35 h/week) showed higher awareness of the latest COVID-19 health online resources. Older practitioners tend to rely more on scientific resources and given that this is a new disease, reliable updated scientific resources will be online, and with long working hours, it is more convenient to seek online resources in their free time [43]. In this study, 88% of the respondents were updated on the MOH guidelines, with no significant difference between the demographic groups, which reflects the exceptional efforts made by the MOH to spread the knowledge and awareness about COVID-19 reaching the majority of health professionals.

“Transmission-based precautions” are a higher level of infection control measures necessary to be taken to prevent the spread of diseases which the routinely “universal precautions” were proven not to be effective in eliminating; such as blood, airborne or droplet transmission [44–46]. The dentists with longer working hours and more years of experience showed significantly higher perceptions of transmission-based precautions. Besides, the longer working experience of practitioners would have subjected them to other infectious diseases and they would most probably have experienced situations that necessitated the adoption of "transmission-based precautions." When asked about the routine use of the universal infection precautions, dentists aged 45 year and older showed significantly higher compliance. Based on other published articles, younger practitioners seem to comply more with infection control measures [47, 48], which is not the case in this study. This finding might be explained by the fact that younger practitioners are more likely to be working longer hours and most probably in more than one clinic, thus focusing on the task or clinical procedure more than the infection control measure itself [5–7].

The last part of the questionnaire asked whether the participants changed their infection routine based on the COVID-19 pandemic facts, where 64% of the participants agreed. Dentists with higher experience and higher qualifications showed significantly higher agreement than others; those two groups were the same groups who showed higher knowledge of the latest news of the COVID-19, which agrees with the concept that awareness significantly affects practice mentioned earlier [39, 49, 50]. Khader et al. have reported that Jordanian dentists showed a limited understanding of extra precautions that needed to be taken against COVID-19 [51]. Duruk et al. investigated the attitude and behavior of Turkish dentists during the pandemic and reported similar results to the finding of this survey regarding changes in infection control measures [24].

To measure the mindset and willingness to adapt new infection control measures and because the use of the N-95 face mask was one of the continuing recommendations to prevent droplet transmission during the pandemic, the participants were asked if they thought the N-95 mask should be routinely worn in dental practice [33]. High agreement was detected by 72% with the number of younger dentists (< 45 years) significantly higher in the agreement response. As mentioned above, other published articles showed that younger practitioners tend to comply more with PPE recommendations [47, 48]. In a similar study in Turkey, only 12% of the participants reported using the N-95 mask [24]. Ahmed et al. reported a higher percentage (up to 89%) in the use of the N-95 mask [23].

### Limitations

Some of the limitations of this study were short period of data collection, keeping in mind the rapid effect of this outbreak on the perception and practices of dental health practitioners; this was reflected on the sample size and number of participants as well as the ability to evaluate the association between the dependent the variables and multiple independent variables. Based on previous survey-based studies that participants tend to complain about lengthy questionnaires, the number of questions in this study was limited to the aspects the authors felt are most important, leaving more areas to be covered further based on the outcome of this study. It is worth mentioning that the survey was conducted while the pandemic was still active. Hence, COVID-19 is considered new and not fully understood or investigated. Updates and recommendations are changed, modified, or added on a day-to-day basis. Therefore, this study sheds light on where we presently stand and cannot be generalized.

## Conclusion

Overall, this study has shown an acceptable level of awareness and preparedness among dental practitioners concerning COVID-19 news, spread, and prevention recommendations, with the highest level of awareness of the MOH guidelines and recommendations. Some dentists reported being unaware of some clinical-patient management procedures to limit the spread of the disease, although the procedures were listed in the MOH guidelines. Thus, dental clinics are required to carry out educational sessions for their dentists and staff on the latest COVID-19 recommendations. Dental clinics are required to closely monitor their staff and dentists to ensure adherence to the MOH guidelines.

## Supporting information

S1 Questionnaire (DOCX)

S1 Data (SAV)
