# Supplementary material for: Pandemic preparedness of dentists against coronavirus disease: A Saudi Arabian experience
Source: PLoS One. 2020 Aug 19;15(8):e0237630. doi: 10.1371/journal.pone.0237630 (PMC7437908; doi:10.1371/journal.pone.0237630)
Supplement: S1 Questionnaire — (DOCX) [file pone.0237630.s001.docx]

Dear Doctor,

Thank you for taking few minutes of your time to respond to this survey about "Attitudes of dental professionals towards infection control post COVID-19 Pandemic."
Your participation will be highly appreciated.

**The following questions will cover demographic information of the participant:**

1. Age
   1. 20–34 yr
   2. 35–44 yr
   3. 45–54 yr
   4. 55–64 yr
   5. >65 yr
2. Gender
   1. Male
   2. Female
3. Qualification
   1. Consultant/Specialist
   2. General dental practitioner
   3. Resident/Graduate research
   4. Dental intern
4. Work experience (in years)
   1. 0–5 yr
   2. 6–10 yr
   3. 11–15 yr
   4. > 16 yr
5. Region of the main job
   1. Eastern
   2. Central
   3. Southern
   4. Western
   5. Northern
6. Work setting of the main job
   1. Private
   2. Governmental
   3. Both (private & governmental)
   4. Academic
7. Working hours per week
   1. 1–19 hr
   2. 20–34 hr
   3. 35–49 hr
   4. 50 + hr

COVID-19 Management in the dental clinics (6 Qs)

1. Does your dental clinic have a work plan (workflow) for COVID-19 patient screening and dental management?
   1. Yes
   2. No
   3. I don’t know
2. Does your dental clinic have a COVID-19 screening questionnaire for patients?
   1. Yes
   2. No
   3. I don’t know
3. Does your dental clinic have an isolation area for suspected COVID-19 patients?
   1. Yes
   2. No
   3. I don’t know
4. Does your dental clinic offer tele-screening for patients prior to their dental visit?
   1. Yes
   2. No
   3. I don’t know
5. Does your dental clinic have an Airborne infection isolation room (AIIR)?
   1. Yes
   2. No
   3. I don’t know
6. Does your dental clinic have an extra-oral suction (vacuum) system?
   1. Yes
   2. No
   3. I don’t know
7. Does your dental clinic offer proper COVID-19 management training sessions?
8. Yes
9. No
10. I don’t know

The following questions deal with dental patients’ reception area: (5 Qs)

1. Are patients required to take their body temperature prior to dental procedure?
   1. Yes
   2. No
   3. I don’t know
2. Are patients required to use an antiseptic mouth rinse prior to dental procedure?
   1. Yes
   2. No
   3. I don’t know
3. Are patients required to wear a face mask in the waiting area?
4. Yes
5. No
6. I don’t know
7. Are patients required to wash/sanitize their hands before going to the waiting area?
8. Yes
9. No
10. I don’t know
11. Is the “social distancing” practiced in the waiting area?
12. Yes
13. No
14. I don’t know

Knowledge, practice and attitude of dentists towards COVID 19 (7 Qs)

1. Are You Updated with the latest news about the spread of the COVID-19 Pandemic?
2. Yes
3. No
4. I don’t know
5. Are You Updated with the latest health online resources for COVID-19?
6. Yes
7. No
8. I don’t know
9. Are You Updated with the Current MOH Guidelines for Cross-Infection Control regarding COVID-19?
10. Yes
11. No
12. I don’t know
13. Before the COVID 19 Pandemic, were you familiar with the “Transmission-Based Precautions” for dental procedures?
14. Yes
15. No
16. I don’t know
17. Before the COVID 19 Pandemic, Did You Routinely Follow Universal Precautions of Infection Control for Every Patient?
18. Yes
19. No
20. I don’t know
21. Did Your infection control routine change after the COVID 19 Pandemic?
22. Yes
23. No
24. I don’t know

If yes,,, please……Specify

1. Do You Think N-95 Mask should be Routinely Worn in Dental Practice as a new precaution?
2. Yes
3. No
4. I don’t know

**Thank you so much for your time**
